# Recent Progress in Photonic Upconversion Materials for Organic Lanthanide Complexes

**DOI:** 10.3390/ma16165642

**Published:** 2023-08-16

**Authors:** Hong-Ju Yin, Zhong-Gui Xiao, Yansong Feng, Chang-Jiang Yao

**Affiliations:** 1 College of Chemistry and Environmental Science, Qujing Normal University, Qujing 655011, China; yinhongju411@163.com (H.-J.Y.); xiaozg1160@outlook.com (Z.-G.X.); 2 State Key Laboratory of Explosion Science and Technology, School of Mechatronical Engineering, Beijing Institute of Technology, Beijing 100081, China

**Keywords:** upconversion luminescence, organic lanthanide complexes, sensitizer, activator, mechanism

## Abstract

Organic lanthanide complexes have garnered significant attention in various fields due to their intriguing energy transfer mechanism, enabling the upconversion (UC) of two or more low-energy photons into high-energy photons. In comparison to lanthanide-doped inorganic nanoparticles, organic UC complexes hold great promise for biological delivery applications due to their advantageous properties of controllable size and composition. This review aims to provide a summary of the fundamental concept and recent developments of organic lanthanide-based UC materials based on different mechanisms. Furthermore, we also detail recent applications in the fields of bioimaging and solar cells. The developments and forthcoming challenges in organic lanthanide-based UC offer readers valuable insights and opportunities to engage in further research endeavors.

## 1. Introduction

Photon upconversion (UC) is a fascinating phenomenon in which a material releases a high-energy photon by continuously absorbing two or more low-energy photons [1,2,3]. Upconversion luminescence (UCL) can efficiently convert near infrared (NIR) light to UV or visible light, which makes it highly promising for various applications in biological NIR imaging probes [4], photogenetic therapy [5], deep lymphoma diagnosis [6], solar cells [7], light-emitting diodes [8], photoredox catalysis [9], etc. Trivalent lanthanide (Ln) complexes are well known for their unique optical properties, such as long-lived excited states, high quantum yields, a large Stokes shift, and sharp and abundant emission lines [10]. Combined with their favorable biodegradability and biocompatibility, organic UC materials hold tremendous potential for solar cells, health-monitoring devices, electronic skin, bioimaging, and optogenetics [11]. UCL research primarily centers around two categories: Ln-doped inorganic materials and organic lanthanide complexes. The Ln-doped inorganic materials typically consist of a host lattice, sensitizer, and activator (e.g., NaYF_4_: Yb/Er) [12,13,14,15,16]. Inorganic upconverted nanoparticles (UCNPs) have issues with surface hardening and repeatability; hence, efforts are being undertaken to minimize these issues by bringing down the size of metal-based systems [17]. Firstly, the size and composition of the organic UC materials could be precisely controlled [18,19]. This control allows for tailoring the properties and performance of the UC materials, enhancing their efficiency and functionality. Secondly, the solubility of the materials can be improved by designing the structure of the ligand, enabling better applicability in vivo. Additionally, the introduction of organic molecular dyes as sensitizers for Ln(III) ion luminescence is expected to enhance molar absorption [18]. Moreover, some organic compounds can be easily and cost-effectively fabricated on flexible substrates at low temperatures, enabling UC in massive thin films. In recent decades, the development of organic UC materials has progressed rapidly. A great number of organic complexes have been prepared by coordinating Ln^3+^ ions with the tri-/poly-dentate helical ligands, and UCL signals have been detected in solution [20,21,22,23,24,25]. The resulting materials come in a variety of forms, including mono-, di-, and tri-nuclear complexes and discrete molecular aggregates. Initially, researchers focused on developing novel organic Ln(III) luminescent materials by modifying the structure of ligands to improve their luminescence intensity in solution and investigating the luminescence mechanism [21,26]. As research in this field has advanced, more attention has been paid to the relationship between structure and luminescence properties, exploring innovative molecular assembly strategies, optimizing ligand structure, improving UCL efficiency, and applying luminescent materials obtained from research in various fields such as biological imaging, luminescence sensing, and solar cells [27,28]. While previous reviews primarily focused on the preparation and study of luminescent materials and luminescent properties [2,29,30,31]. This review aims to summarize the recent progress ever made in the fields of luminescent organic complex materials based on Ln(III) ions. This paper begins by introducing the composition and typical luminescence mechanism of organic UCL materials, followed by a review of the latest developments in organic UCL materials research. The potential applications of organic UCL materials in biological imaging and solar cells are discussed. Finally, the review concludes with the potential prospects and challenges for further development in this field. 

## 2. The Basic Concept of Organic Lanthanide-Based UC

Organic UC materials mainly include two parts: an activator (accepter) and a sensitizer (donor). The UC phenomena are evoked by sensitizer-activator interactions. 

### 2.1. Sensitizer

The role of the sensitizer is to absorb light from the pumping source and requires a relatively large absorption cross section (σ_abs_), usually at least one order of magnitude higher than the associated activator. Until now, the three most commonly used sensitizers were Yb^3+^, Nd^3+^, and Er^3+^. The sensitizer Yb^3+^ directly interacts with the activator (e.g., Er^3+^, Tm^3+^, and Ho^3+^). While the sensitizers Nd^3+^ and Er^3+^ need the cooperation of Yb^3+^ to complete the whole process. 

### 2.2. Activator

The activator is used to achieve UCL by using its stepped energy levels. Because each activator has its own unique energy level structure, its UCL shows sharp emission peaks with a differentiable spectroscopic fingerprint. Most of the Ln^3+^ ions can produce UCL, such as Pr^3+^, Nd^3+^, Sm^3+^, Eu^3+^, Gd^3+^, Tb^3+^, Dy^3+^, Ho^3+^, Er^3+^, and Tm^3+^. Until now, Er^3+^ ions have been the most efficient activators for green and red UCL. Er^3+^ ions can also be sensitizers because of their nearly perfect ladder-like energy levels. More interestingly, Er^3+^ ions occur simultaneously as sensitizers and activators and can produce strong UCL through self-sensitization. 

### 2.3. The Mechanisms of Upconversion Luminescence

**Figure 1:**
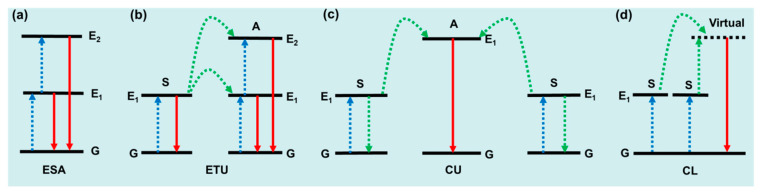
Schematic linear upconversion mechanisms operating in isolated molecules: (**a**) ESA, (**b**) ETU, (**c**) CU, and (**d**) CL. S, sensitizer; A, activator; G, ground state; E_1_/E_2_, excited state; blue, linear excitation; green, energy transfers; red, upconverted emission [32].

UCL mechanisms include excited state absorption (ESA), energy transfer upconversion (ETU), cooperative luminescence (CL), and cooperative upconversion (CU), as shown in Figure 1. ESA describes the simplest UC process, where the emission is caused by sequential absorption within the energy levels of a given ion (Figure 1a). In principle, ESA could occur in many of the Ln^3+^ ions, such as Er^3+^, Dy^3+^, Tm^3+^, Ho^3+^, and Pr^3+^. ESA only plays an important role when the doping concentration of Ln^3+^ is relatively low, and materials based on the ESA mechanism are limited by low absorption and low UC emission intensity. ETU is recognized as the dominant mechanism in the most efficient UC system. As shown in Figure 1b, UC can be realized from the activator (A) in a more excited state, while the sensitizer (S) returns to its ground state. Note that excited states sometimes transition to lower energy levels beyond the ground state, and this particular ETU process is also called cross relaxation (CR). Compared with ESA, ETU allows a much higher doping concentration of Ln^3+^, which offers a much stronger absorption ability to the material. The UC efficiency of ETU is usually more than two orders of magnitude higher than that of ESA. Cooperative upconversion (CU) is also known as cooperative sensitization upconversion (CSU) in some literature. As shown in Figure 1c, one activator (A) ion receives energy from two nearby excited sensitizer (B) ions at the same time, and the excited activator relaxes back to its ground state by producing a UC photon. The rare cooperative luminescence (CL) lacks an activator-centered excited relay and is replaced by a virtual emissive state [32], as shown in Figure 1d. 

## 3. Organic Lanthanide-Based UC Materials

**Scheme 1:**
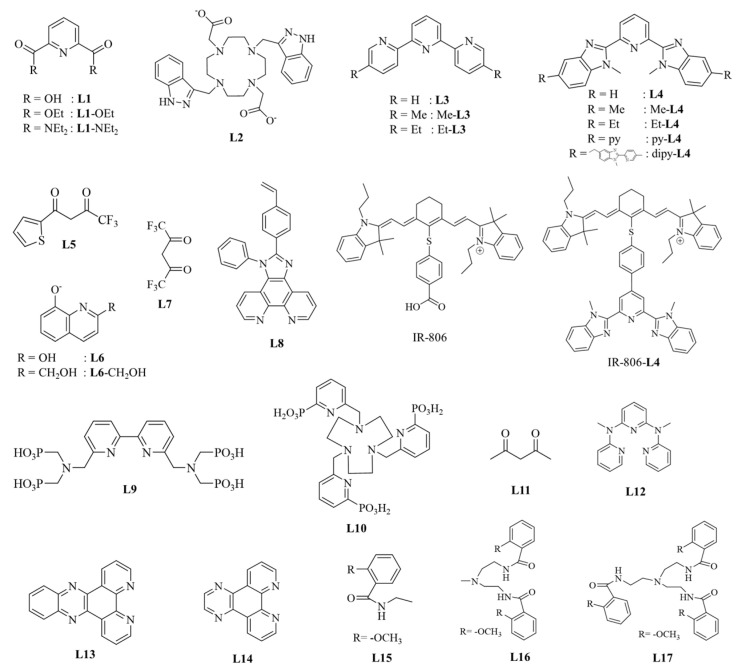
Diagram of ligand structures in organic complexes of four different mechanisms.

The ligand structures of the Ln(III) UC complexes reviewed in this paper are shown in Scheme 1. The latest research on UCL of organic complexes based on ESA, ETU, CL, and CU mechanisms will be discussed below. The UCL data on organic complexes are summarized in Table 1. 

### 3.1. Upconversion Based on the ESA Mechanism

**Figure 2:**
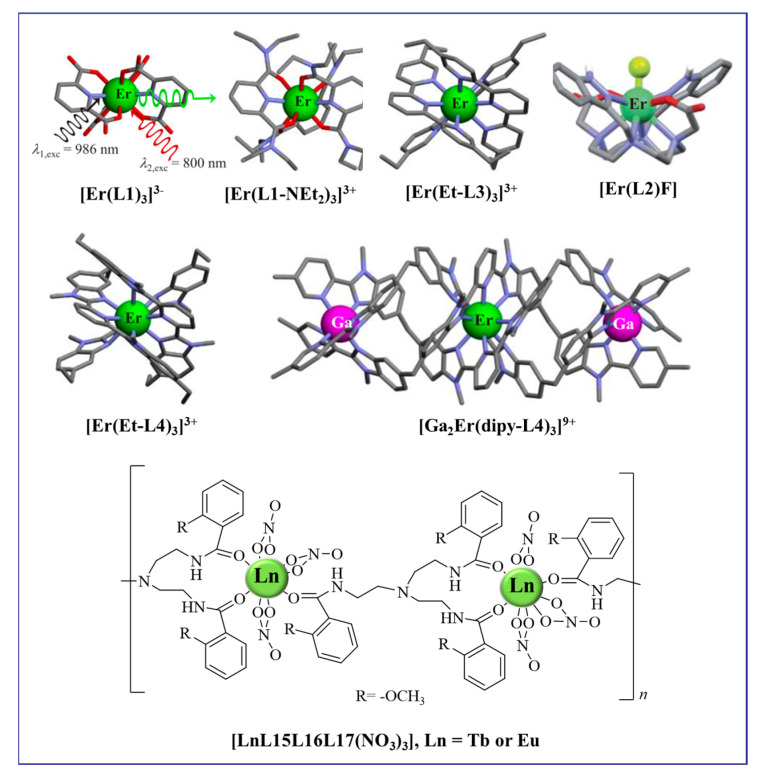
Diagram of organic complexes structures based on the ESA mechanism.

Mononuclear rare earth complexes often exhibit UCL based on the ESA mechanism. As early as the turn of the century, the aqueous solution of [Er(**L1**)_3_]^3−^ was found to exhibit weak NIR to visible UCL under the excitation of a high-power dual-mass laser pump (Figure 2) [33]. By effectively shielding the metal Ln center from interactions with the high-energy oscillators of ligands or solvent molecules, the lifetime of the intermediate excited state can be prolonged, enabling the detection of UC signals from individual Er central complexes under appropriate excitation power. In 2016, Charbonnière and co-workers suggested that the UC visible signal of the monomeric complex [Er(**L2**)F] in deuterated water might be attributed to a single-center ESA mechanism upon 980 nm excitation (*p* = 10 W·cm^−2^) [22]. The coordination of the fluoride anions to saturate the Er coordination sphere enhances the UCL. As shown in Figure 2, the elaborately designed polyaromatic tridentate ligands possess greater complexities and sizes. After forming a single-center complex with an Er ion, ESA UC can be realized at reasonable excitation intensities. When excited at 801 nm (Er, ^4^I_9/2_ ← ^4^I_15/2_ transition) with *p* = 25 W·cm^−2^, the triple-helical complexes [Er(**L1**)_3_]^3−^ [33], [ErL_3_]^3+^ (L = R-**L1**, R-**L3**, and R-**L4**) [23,24], and [GaErGa(dipy-**L4**)_3_]^9+^ [25] exhibited two-photon green UCL (^4^S_3/2_ → ^4^I_15/2_) and one-photon down-conversion luminescence in acetonitrile solution at room temperature. The quantum yields, although low, can be adjusted with structural modifications (Table 1). In 2018, Piguet and co-workers reported an unprecedented related UCL emission from a single nine-coordinate Er complex [Er(Et-**L4**)_3_]^3+^ upon 800 nm excitation via the ESA mechanism [24]. In addition to discrete molecular upconversion, back in 2004, Cheah and co-workers demonstrated a three-photon upconversion process based on organic-lanthanide (Ln = Tb, Eu) polymers. The structure of the polymeric chain [Ln**L15L16L17**(NO_3_)_3_] is shown in Figure 2. At 845 nm excitation, Tb and Eu polymer chains exhibited green and red light, respectively, based on the ESA mechanism [34]. 

Effectively protecting metal centers from non-radiative inactivation sources located on ligands (such as fluorination or deuteration of these surrounding ligands) represents a promising strategy for enhancing the lifetime of the intermediate excited state, particularly in the case of Tm and Er activators [35]. 

### 3.2. Upconversion Based on the ETU Mechanism

In order to efficiently populate the activators excited states, organic dyes or metal sensitizers (mostly Yb^3+^ and Nd^3+^) are commonly used for energy absorption and to transfer energy to activators through the ETU mechanism [18,36,37,38,39,40]. 

**Figure 3:**
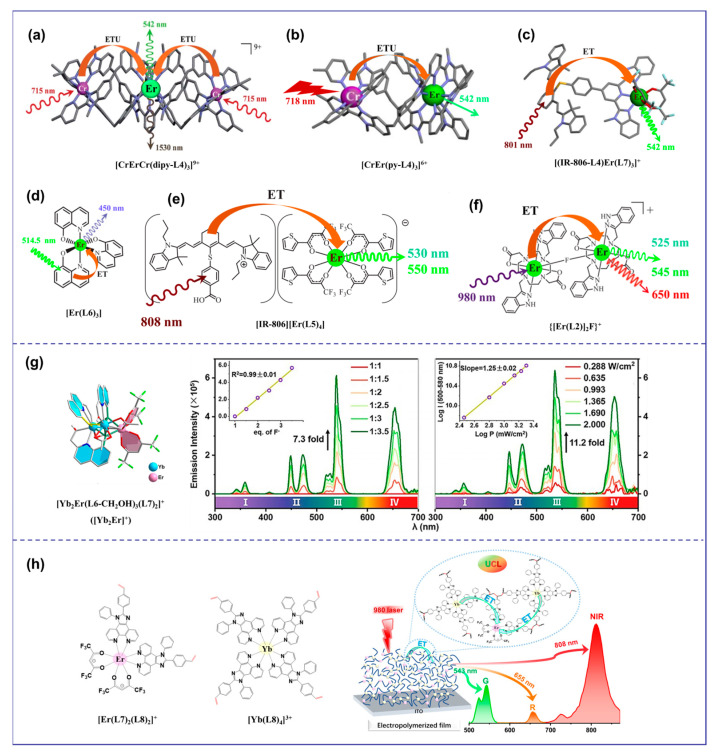
Diagram of organic complexes structures based on the ETU mechanism. (**a**), the structure diagram of the trinuclear complex [CrErCr(dipy-**L4**)_3_]^9+^ [20]; (**b**), the structure diagram of [CrEr(py-**L4**)_3_]^6+^ [21]; (**c**), the structure diagram of [(IR-806-**L4**)Er(**L7**)_3_]^+^ [37]; (**d**), the structure diagram of [Er(**L6**)_3_] [42]; (**e**), the structure diagram of [IR-806][Er(**L5**)_4_] [18]; (**f**), the structure diagram of {[Er(**L2**)]_2_F}^+^ [22]; (**g**), the structure diagram of the discrete bimetallic [Yb_2_Er]^+^ and the UCL intensity (545 nm) of [Yb_2_Er]^+^ in CH_2_Cl_2_ dependent on the quantity of [Bu_4_N]F under 2 Wcm^−2^ (**left**) and dependent on the quantity of [Bu_4_N]F under 2 Wcm^−2^ (**right**) [45]; (**h**), the structure diagram of [Er(**L7**)_4_(**L8**)_2_]^+^ and [Yb(**L8**)_4_)]^3+^ and the emission spectrum of polymer films [46]).

Discrete molecular UC using the ETU mechanism can be traced back to the trinuclear complex [CrErCr(dipy-**L4**)_3_]^9+^ (S = Cr, A = Er) (Figure 3a) [20]. The UC’s efficiency was significantly enhanced through ETU. For the simplest case of the S/A pair found in [CrEr(py-**L4**)_3_]^6+^ (S = Cr, A = Er) (Figure 3b), the efficiency of the ETU mechanism depends on the competition between the excitation process of the second sensitizer and the relaxation rate of the intermediate excited state of the activator [21]. Compared with [Er(Et-**L4**)_3_]^3+^ (*ϕ*_up_(ESA) = 1.7 × 10^−9^), the predicted upconverted quantum efficiency (*ϕ*_up_(ETU) ≈ 3 × 10^−15^) was 6 orders of magnitude lower and therefore negligible at *p* = 38.2 W·cm^−2^. Antenna dyes with wide absorption spectra and high absorption coefficients have been used to overcome the limited absorption of lanthanide ions [41]. These dyes, when used as light collection antennas on the surface of UCNPs, dramatically enhance UCL intensity (about factor 3300) [41]. Moreover, an organic antenna-sensitized Er-based complex demonstrated molecular photon-upconverted luminescence in a solution at room temperature [18]. By replacing the less efficient sensitizer Cr^3+^ in [CrEr(py-**L4**)_3_]^6+^ with a NIR-absorbing cyanine dye, we cured the inherent low-efficient ESA mechanism with a powerful indirect sensitization by the ETU mechanism, which improved the upconverted quantum efficiency (*ϕ*_up_(ETU) = 1.9 × 10^−10^) of [(IR-806-**L4**)Er(**L7**)_3_]^+^ at 801 nm excitation (Figure 3c) [37]. This strategy had also been employed to detect UC signals in organic films containing [Er(**L6**)_3_] complexes (Figure 3d) [42]. Subsequently, the discrete molecular entities showing UC using the ETU mechanism were extended to the formation of ion [IR-806][Er(**L5**)_4_] pairs in non-polar organic solvents (Figure 3e) [18] and fluoro-bridged dimers {[Er(**L2**)]_2_F}^+^ in water (Figure 3f) [22]. Heterometallic sensitizer-acceptor molecular blocks (Yb*_n_*Er) are the most promising candidates for molecular UC of organic complexes [26]. The discrete polymetallic complexes Yb*_n_*Er can not only reduce the molecular size but also introduce sensitizers Yb^3+^ to sensitize the Er^3+^ center [20,22,43], compared with the mononuclear Er^3+^ complex [24]. In 2017, UC signals were observed in the organic complex Er_0.67_Yb_1.33_[(CF_3_)_2_CHO]_9_ due to intramolecular energy transfer from Yb^3+^ to Er^3+^ [39]. The overabundance of Yb^3+^ boosted the probability of a Yb→Er energy transfer, and it was found that raising the Yb/Er ratio can significantly improve the UC efficiency, as demonstrated in the nanoscale [Er_2_Yb_13_] molecular cluster-aggregate [44]. However, it is extremely difficult to arbitrarily adjust the Yb/Er ratio in a particular discrete molecular or construct a free doping organic system like inorganic UCNPs (e.g., NaYF_4_: Yb, Er). Consequently, developing organic UC systems with optional Ln elements and adjustable sensitizer/activator ratios is an attractive but challenging task. Recently, Wang et al. [45] found that the NIR downshift luminescence of discrete bimetallic [Yb_2_Er]^+^ (Figure 3g) with metal distances of approximately 3.7 Å, transformed into competitive UCL upon irradiation by a 980 nm laser under an extremely low excitation power (0.288 W·cm^−2^) with the aid of fluoride ions. In 2023, we proposed a novel organic UC system and a polymerization approach that connected sensitizer Yb^3+^ with activator Er^3+^ in one polymer through chemical polymerization and electrochemical polymerization for the first time [46]. Under the irradiation of a 980 nm laser, all the polymers [polyEr(**L7**)_4_(**L8**)_2_-Yb(**L8**)_4_] (Figure 3h) exhibited dual visible and NIR UC signals in solution or solid state, and the UCL properties of the polymers could be significantly enhanced by merely adjusting the Yb/Er ratio.

### 3.3. Upconversion Based on the CU Mechanism

**Figure 4:**
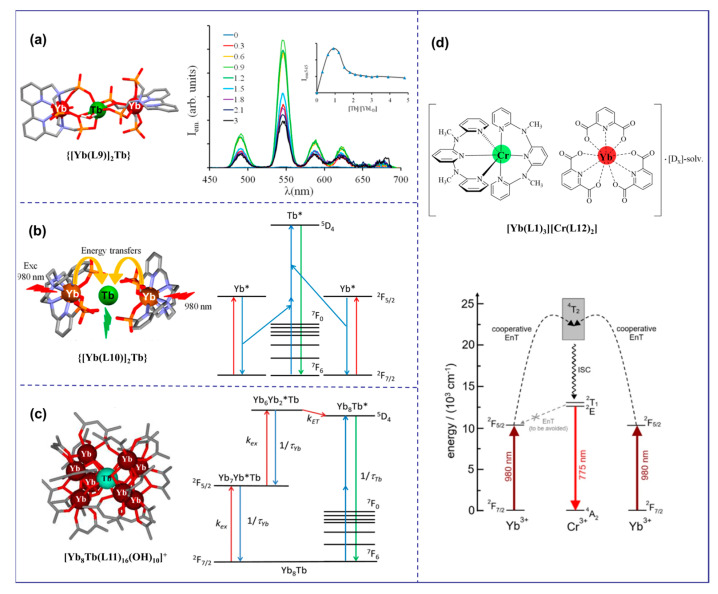
Diagram of organic complexes structures based on the CU mechanism. (“*” represents a species in an excited state; (**a**), the structure diagram of {[Yb(**L9**)]_2_Tb and the emission spectrum at 980 nm excitation [48]; (**b**), the structure diagram of {[Yb(**L10**)]_2_Tb} and the luminous mechanism [43]; (**c**), the structure diagram of [Yb_8_Tb(**L11**)_16_(OH)_10_]^+^ and the luminous mechanism [49]; (**d**), the structure diagram of [Yb(**L1**)_3_][Cr(**L12**)_2_] and the luminous mechanism [50]).

The cooperative upconversion (CU) mechanism is a double-operator process that occurs when there are no available intermediate excited states that are compatible with ETU mechanisms [47]. The CU mechanism is considered only when ETU cannot occur, but it is significantly less efficient—several orders of magnitude less efficient than the ETU mechanism [30]. Thus, CU becomes interesting for sensitizing Ln ions Eu^3+^, Gd^3+^, and Tb^3+^, which exhibit impressive intrinsic Eu(^5^D_0_ → ^7^F_J_), Tb(^5^D_4_ → ^7^F_J_), and Gd(^6^P_7/2_ → ^8^S_7/2_) emission quantum yields, respectively. As shown in Figure 4a, {[Yb(**L9**)]_2_Tb_x_} (*x* = 1 to 3) complexes were prepared in deuterated water for the first time by combining Yb^3+^ sensitizers with Tb^3+^ activators using a phospho-based bipyridine ligand by a step-by-step coordination mechanism. Upon NIR excitation at 980 nm, an unprecedented UC of Yb-sensitized Tb luminescence was observed, resulting in the characteristic green emission of Tb(^5^D_4_→^7^F_5_) (*ϕ*_up_(CU) = 1.4 × 10^−8^ at *p* = 10.3 W·cm^−2^) [48]. This two-photon UC mechanism can be explained by the CU mechanism, where the first Yb^3+^ ion transfers energy to the second Yb^3+^ ion, which then collaboratively excites the Tb^3+^ ion. A similar phenomenon was observed in deuterated water with {[Yb(**L10**)]_2_Tb_x_} mixtures (x = 1, 2; *ϕ*_up_(CU) = 1.4 × 10^−8^ at *p* = 2.86 W·cm^−2^ Figure 4b) [43]. Recently, the Charbonnière group had observed effective cooperative UC photosensitization in a Tb-doped heteronuclear complex [Yb_8_Tb(**L11**)_16_(OH)_10_](OH) (Figure 4c) at low concentrations, and its UC efficiency (*ϕ*_up_(CU) = 1 × 10^−7^ at *p* = 2.86 W·cm^−2^) was more than one order of magnitude more efficient than previous molecular or supramolecular UC probes [49]. Additionally, CU was reported in solid hybrid materials, such as the cocrystallized [YbL_3_]_0.7_[TbL_3_]_0.3_ coordination complex (L = perfluorimide diphosphate ligand) and in the ionic [Yb(**L1**)_3_][Cr(**L12**)_2_] salt (Figure 4d) [50].

### 3.4. Upconversion Based on the CL Mechanism

**Figure 5:**
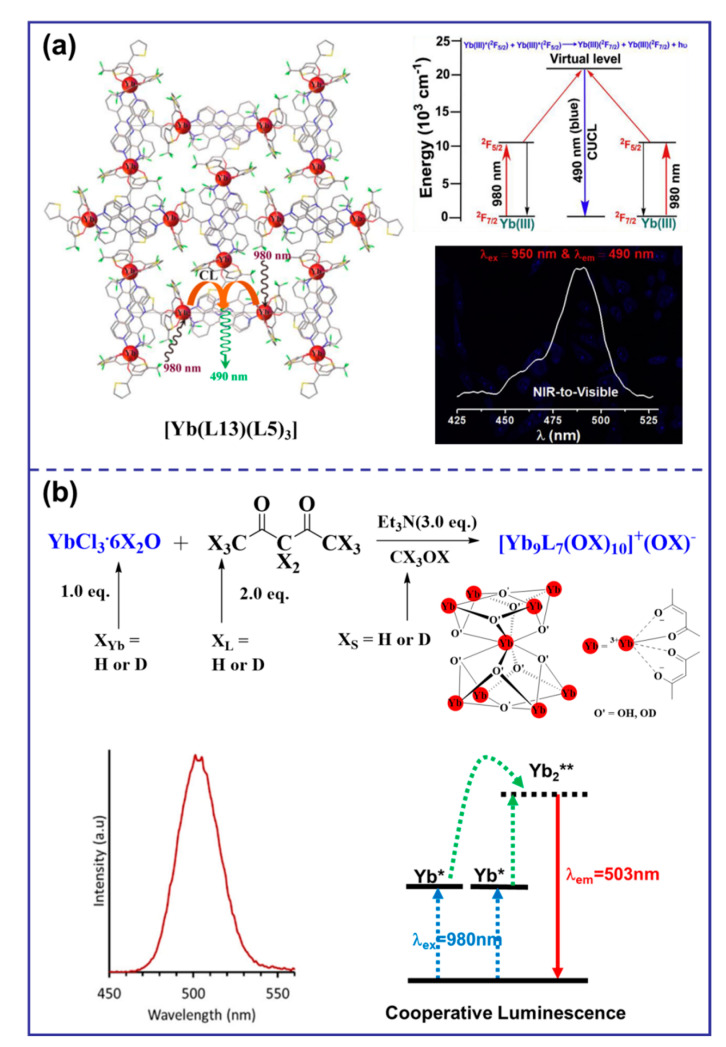
Diagram of organic complexes structures based on the CL mechanism. (“*” represents a species in an excited state; “**” represents a species in a virtual excited state; (**a**), the structure diagram of [Yb(**L13**)(**L5**)_3_] and the emission spectrum at the 950 nm excitation and the luminous mechanism [51]; (**b**), the structure diagram of [Yb_9_**L7**(OX)_10_]^+^ and the emission spectrum at 980 nm excitation and the luminous mechanism [19]).

Cooperative luminescence (CL) is a rare UC phenomenon that occurs when the excitation relay with the activator as the center is absent, resulting in its replacement by a virtual emission state [32]. In a case reported in 2019, solid salt [Yb(**L13**)(**L5**)_3_] exhibited visible blue light at 490 nm upon NIR excitation at 980 nm (Figure 5a), which made them promising candidates for NIR-to-visible or NIR-to-NIR cellular imaging probes [51]. In 2021, a surprising case of molecular solution co-luminescence (CL) UC was observed due to the excited states of Yb^3+^. When the [Yb_9_**L7**(OX)_10_]^+^ complex was excited by the laser at 980 nm, a symmetric emission band with a maximum at approximately 503 nm was observed in pure CD_3_OD, which was attributed to the CL originating from Yb clusters (Figure 5b) [19].

**Table 1:** UCL data of organic Ln complexes in recent years.

Complex	λ_ex_ (nm)	λ_em_ (nm)	*p* (W·cm^−2^)	*ϕ*_up_	τ (μs)	Ref.
[Er(**L1**)_3_]^3−^	986	542	10^9^	/	/	[33]
[Er(**L2**)F]	980	520–650	10	/	/	[22]
[Er(**L1**-NEt_2_)_3_]^3+^	801	522–542	25	1.9 × 10^−10^	3.07	[23]
[Er(Et-**L3**)_3_]^3+^	801	522–542	25	5.5 × 10^−11^	1.88	[24]
[Er(Et-**L4**)_3_]^3+^	801	522–542	25	1.7 × 10^−9^	5.56	[24]
[Ga_2_Er(dipy-**L4**)_3_]^9+^	801	522–542	25	1.7 × 10^−9^	Er: 4.03	[25]
[CrEr(py-**L4**)_3_]^6+^	718	522–542	38.2	5.3 × 10^−8^	Er: 4.6	[21]
[CrErCr(dipy-**L4**)_3_]^9+^	718	522–542	38.2	5.8 × 10^−8^	Er: 3.6	[20]
[(IR-806-**L4**)Er(**L7**)_3_]^+^	801	522–542	1.4	1.9 × 10^−10^	/	[37]
[Er**L6**]	514	450	/	/	/	[42]
[Yb_2_Er]^+^	980	545–655	2	0.0182	Er: 94.4	[45]
[polyEr(**L7**)_4_(**L8**)_2_-Yb(**L8**)_4_	980	543–808	5	/	Er: 123	[46]
{[Yb(**L9**)]_2_Tb}	980	485–680	10.3	1.4 × 10^−8^	/	[48]
{[Yb(**L10**)]_2_Tb}	980	480–650	2.86	1.4 × 10^−8^	Tb: 1.46 × 10^3^	[43]
[Yb_8_Tb(**L11**)_16_(OH)_10_]^+^	980	480–650	2.86	1.0 × 10^−7^	Tb: 1.12 × 10^3^	[49]
[Yb(**L1**)_3_][Cr(**L12**)_2_]	976	780	67	/	Cr: 380	[50]
[Yb(**L13**)(**L5**)_3_]	980	490	/	/	15.7	[52]
[Tb**L15L16L17**(NO_3_)_3_]	845	450–700	/	/	/	[34]
[Eu**L15L16L17**(NO_3_)_3_]	845	600–730	/	/	/	[34]

## 4. Applications

In recent decades, organic Ln(III) complexes based on UCL have been rapidly developed, and various Ln(III) complexes with different mechanisms have been prepared. These materials not only have excellent luminescence properties derived from Ln(III), but also exhibit favorable solubility, customizable particle size, and excellent film-forming characteristics, which make them have certain application potential in the fields of bioimaging and solar cells. 

### 4.1. Bioimaging Application

NIR imaging has gained significant interest in the biomedical industry due to its advantages over traditional visible-light imaging [53]. Longer wavelength stimulation allows for improved tissue penetration by minimizing self-absorption and light scattering caused by the heterogeneity of human tissue [52]. In the last decades, numerous Ln complexes have been investigated as luminescence probes or imaging agents for bio-applications [54,55,56]. While several reviews on this subject have been published elsewhere [57,58,59,60,61,62], optical imaging has emerged as a precise method for observing cellular metabolism and physiological behavior [63]. For effective biological imaging, Ln(III) complexes require a long luminescence lifetime, bright luminescence, a high quantum yield, significant photostability and thermal stability, as well as a large Stokes shift. However, most Ln(III) complexes faced limitations in living cell bioimaging due to UV-radiation damage, limited exposure time, photobleaching, local bioluminescence scattering, and poor tissue penetration. UC materials offer notable advantages in the field of bioimaging, such as increased excitation penetration depth in vivo, eliminating autofluorescence, and preventing irradiated tissue damage. These materials efficiently convert low-energy, deep-penetrating light (longer wavelength) to higher-energy light (shorter wavelength). In this section, the overall research on bioimaging applications in vivo of organic Ln(III) complexes based on UC is summarized.

**Figure 6:**
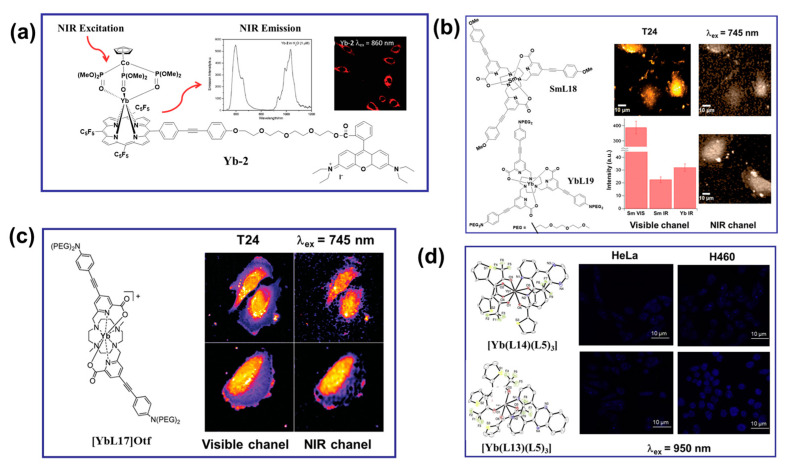
(**a**) Molecular structure, emission spectrum, and two-photon (λ_ex_ = 860 nm) excitation-induced in vitro imaging in HeLa cells (40× magnification, λ_em_ = 500−800 nm) of the porphyrin-based **Yb**−**2** complex [67]. (**b**) The structures of the Sm and Yb complexes used for bioimaging experiments. Visible and NIR microscopy images of T24 cells fixed with PFA under laser irradiation at λ_ex_ = 745 nm by using the [Sm**L15**] emission in the visible channel and the NIR channel (the first row); for comparison, the [Yb**L16**] emission in the NIR channel is also given (bottom right corner). Average intensities and associated standard deviations (bars) measured in central regions of interest (ROIs) of cells (bottom left corner) [69]. (**c**) The structure of the [Yb**L17**]Otf used for bioimaging experiments. 2P−imaging of PFA−fixed T24 cells stained with [Yb**L17**]Otf (c = 10^−5^ mol·L^−1^, 2P excitation at λ_ex_ = 745 nm) using the custom-made NIR-to-NIR microscope. Detection in visible channel (<740 nm) and the NIR spectral (>840 nm) [70]. (**d**) MCLSFM images of the HeLa and H460 cells treated with complexes [Yb(**L14**)(**L5**)_3_] (the first row) and [Yb(**L13**)(**L5**)_3_] (the second row). NIR-to-visible cooperative upconversion emission shows nuclear and cytosolic localization of the complexes. λ_ex_ = 950 nm, and λ_em_ = 490 nm [51].

NIR radiation Yb(III) complexes have garnered significant attention in the field of multiphoton cell imaging. Despite their low luminescent quantum yield, Yb(III) complexes allowed excitation and emission in both the NIR-to-visible and NIR-to-NIR configurations [64,65]. NIR excitation by UC minimizes the scattering and autofluorescence from biological media, enhances the S/N ratio, and enables deep tissue penetration for imaging and bioanalysis [64,65,66]. In 2011, Wong and co-workers reported an Yb complex with exceptional luminescence properties in water and a remarkable two-photon cross-section. Interestingly, this complex was successfully utilized as a biological probe for imaging HeLa cells by two-photon microscopy, operating in the classic NIR-to-visible configuration for detection of residual ligand central emission (Figure 6a) [67]. Complexes [Yb**L16**] were also identified as a 2P NIR biological probe with an emission wavelength of 980 nm, demonstrating a promising thick-tissue imaging probe [68]. In 2015, Maury et al. synthesized a samarium complex [Sm**L15**] structurally similar to [Yb**L16**]. The stained fixed cells could be imaged in the visible light and NIR spectrums. Under the same conditions, the 2P images obtained by [Sm**L15**] in the NIR spectrum showed a similar distribution to that obtained with [Yb**L16**] (Figure 6b) [69]. As shown in Figure 6c, in 2017, Maury et al. prepared Yb(III) complexes ([Yb**L17**]Otf) based on the dimethyl cycline macrocyclic ligand and obtained high-quality images in both the classic NIR-to-vis configuration and the more challenging NIR-to-NIR configuration [70]. Yb(III) complexes offer potential for biological imaging and serve as effective light therapy agents for deep tissues due to their NIR excitation and emission and unique cooperative upconversion luminescence properties. In 2019, Patra and co-workers designed double-sensitized (**L14**/**L13** and **L5**) Yb(III) complexes to modulate the desirable optical properties in the NIR region for bioimaging (Figure 6d) [51]. The cytosolic and nuclear localizations were monitored by utilizing their unique and intrinsic cooperative upconversion luminescence of Yb(III) ions in the NIR-to-visible region (λ_ex_ = 950 nm) and multiphoton excitation (λ_ex_ = 750 nm) using confocal fluorescence microscopy in HeLa and H460 cancer cells. The cellular uptake studies clearly demonstrated the cytoplasmic and nuclear localization of the complex. The NIR cytotoxicity of [Yb(**L13**)(**L5**)_3_] upon continuous 980 nm irradiation by a laser demonstrated its potential for PDT. These results provided an intelligent strategy for the development of light-responsive, highlight-stable Yb(III) probes for NIR therapy applications in biologically transparent phototherapeutic windows [51]. 

Bioavailability and cytotoxicity are important criteria for evaluating potential applications of bioimaging probes [71,72,73]. The advantages of organic lanthanide complexes over inorganic nanomaterials and organic molecules include tunable metal toxicity and easy modification [74]. For instance, when utilized in biological imaging, numerous visible/NIR emissive lanthanide complexes showed low toxicity [75,76,77,78,79,80,81]. Significant differences in the sensitivities of the applied cells as well as in the cytotoxic effects of various lanthanides on the same cell line were observed. By studying the interaction of Eu(III) with FaDu cells, Sachs and co-workers [79] found that the cytotoxicity of Eu(III) on FaDu cells was correlated to their cellular uptake, both being mainly concentration-dependent and only slightly time-dependent. They demonstrated that the cytotoxicity of Eu(III) on FaDu cells was mainly controlled by insoluble species. Additionally, organic lanthanide complexes can be structurally modified to simply increase the biocompatibility of the complexes and control the bioavailability of probe molecules [74]. Although bioimaging applications of organic lanthanide-based UC materials have been reported in a small number of cases, in-depth research concerning the impact of the lanthanide speciation on their cytotoxic behavior and bioavailability is still lacking. 

### 4.2. Solar Cell Application

Recently, upconversion has gained recognition as a promising field in “third–generation photovoltaics”, which aims to overcome the efficiency limits of traditional single-threshold photovoltaic devices (Shockley–Queisser limit [82]) [83,84]. Silicon solar cells are crucial for renewable energy sources [85], and the use of Ln UC materials has shown potential due to their ability to absorb sunlight for silicon solar cells [86]. Planar luminescent layers embedded with LnCs in polymer substrates such as PVA, PMMA, and EVA were often used as spectral converters for silicon solar cells to improve PCE [87]. In this context, Wang and co-workers [88] developed a highly stable luminescent copolymer film consisting of the Eu^III^ complex [Eu(CTAC)(ND)_4_, CTAC = hexadecyl trimethyl ammonium chloride, ND = 4-hydroxy-2-methyl-1,5-naphthyridine-3-carbonitrile] and EVA. Coating the surface of a large-area polycrystalline silicon solar cell (110 cm^2^) with a stable luminescent film resulted in an increased PCE of 15.06 to 15.57. The UC strategy has been proven to prolong the NIR response of the PVSCs by converting NIR light into visible light [89,90], which is subsequently reabsorbed by the perovskite photoactive layer to generate additional photocurrent in the PVSCs. Unlike the high light intensities required to integrate Ln(III)-based UCNPs into PVSCs, the TTA UC process in organic semiconductors can be efficient even at subsolar photon fluxes due to the energy stored in the long-lived triplet states. However, to our knowledge, the UCL of organic Ln(III) complexes has been rarely reported in PVSCs. 

**Figure 7:**
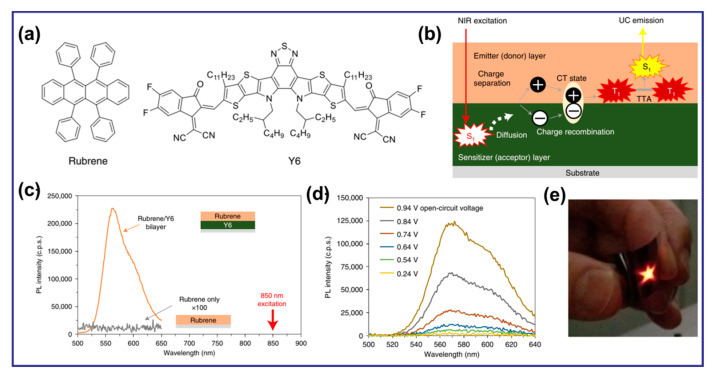
(**a**) Chemical structures of Rubrene and Y6. (**b**) The UC mechanism proposed in this study with bilayer films of sensitizer and emitter. (**c**) UC emission from the rubrene/Y6 bilayer (orange) and pristine rubrene (grey) films irradiated by an 850-nm single-color LED at a power density of 101 mW·cm^−2^. For clarity, the PL intensity of the pure rubrene film was magnified by a factor of 100. (**d**) UC emission spectra of a rubrene/ITIC-Cl bilayer photovoltaic device under an applied bias with incident light from a 750-nm single-color LED with a power density of 78.4 mW·cm^−2^. (**e**) Photographs of UC emission by star-patterned NIR LED irradiation (750 nm, 71.7 mW·cm^−2^) on a flexible thin film [93].

In addition, recent studies have highlighted the potential of organic–inorganic fluorescent materials containing Ln^III^ for use in LED devices as light-converting materials [91,92]. However, there is limited research on the utilization of Ln organic complexes for UCL in LEDs. Figure 7 illustrates a noteworthy UC system with heterojunctions of organic semiconductors, which can effectively convert NIR light to visible light on flexible organic films under the excitation of weak LEDs. Particularly, the bilayer film exhibited efficient UC, producing bright yellow emission visible to the naked eye when excited by NIR LEDs arranged in a star pattern [93]. 

## 5. Summary and Perspective

Ln(III) complexes with long-lived excited states and well-defined scales offer the best prospects for UCL in discrete molecules. Using organic ligands with an adjustable structure and host-guest assembly strategy, organic lanthanide complex materials have the advantages of controllable size, good stability, adjustable solubility, strong machinability, and excellent luminescence properties, which are conducive to the full development of lanthanide complexes in various practical applications. This paper describes the mechanism of UCL based on organic lanthanide complexes and reviews the recent UCL properties of organic Ln(III) complex materials based on four well-known UC mechanisms, including ESA, ETU, CU, and CL. The application prospects of the material in bioimaging and solar cell fields are discussed. 

Despite significant advancements in the field of organic metal UCL, there remain several challenges that require further investigation and exploration. Future research efforts should focus on the following aspects: (i) Exploring new scalable UC systems, including thin-film systems, with new approaches and architectures. Thin-film systems offer potential advantages such as improved light absorption and integration with various device platforms. (ii) Developing new material systems with unique and advanced functions. This may involve the development of materials that exhibit dynamic response performance, enabling their application in fields beyond traditional sensing and imaging. (iii) Expanding the use of organic metal UCL in vivo by increasing complex solubility through ligand modifications. (iv) Leveraging near-infrared-excited organic UCL materials for biological applications to improve the penetration properties. Further research should focus on optimizing and tailoring these materials for improved biological imaging, sensing, and therapeutic applications. 

Addressing these challenges and pursuing advancements in these areas will contribute to the continued progress and broadening of applications for organic metal UCL materials in various fields. 

## Data Availability

The data involved in this manuscript can be found in the corresponding references in the original data.
